# Classification of vasovagal syncope from physiological signals on tilt table testing

**DOI:** 10.1186/s12938-024-01229-9

**Published:** 2024-03-30

**Authors:** Mahbuba Ferdowsi, Ban-Hoe Kwan, Maw Pin Tan, Nor’ Izzati Saedon, Sukanya Subramaniam, Noor Fatin Izzati Abu Hashim, Siti Sakinah Mohd Nasir, Imran Zainal Abidin, Kok Han Chee, Choon-Hian Goh

**Affiliations:** 1 Department of Mechatronics and Biomedical Engineering, Lee Kong Chian Faculty of Engineering and Science, Universiti Tunku Abdul Rahman, 43000 Kajang, Malaysia; 2 Centre for Healthcare Science and Technology, Universiti Tunku Abdul Rahman, 43000 Kajang, Malaysia; 3 Ageing and Age-Associated Disorders Research Group, Department of Medicine, Faculty of Medicine, Universiti Malaya, 50603 Kuala Lumpur, Malaysia; 4 Cardiorespiratory Laboratories, Universiti Malaya Medical Center, 50603 Petaling Jaya, Malaysia; 5 Department of Cardiology, Department of Medicine, Faculty of Medicine, Universiti Malaya, 50603 Kuala Lumpur, Malaysia

**Keywords:** Syncope, Head-up tilt test, Machine learning, Explainable artificial intelligence, Partial dependence plot

## Abstract

**Background:**

The diagnostic test for vasovagal syncope (VVS), the most common cause of syncope is head-up tilt test (HUTT) assessment. During the test, subjects experienced clinical symptoms such as nausea, sweating, pallor, the feeling of palpitations, being on the verge of passing out, and fainting. The study's goal is to develop an algorithm to classify VVS patients based on physiological signals blood pressure (BP) and electrocardiography (ECG) obtained from the HUTT.

**Methods:**

After 10 min of supine rest, the subject was tilted at a 70-degree angle on a tilt table for approximately a total of 35 min. 400 µg of glyceryl trinitrate (GTN) was administered sublingually after the first 20 min and monitoring continued for another 15 min. Mean imputation and K-nearest neighbors (KNN) imputation approaches to handle missing values. Next, feature selection techniques were implemented, including genetic algorithm, recursive feature elimination, and feature importance, to determine the crucial features. The Mann–Whitney U test was then performed to determine the statistical difference between two groups. Patients with VVS are categorized via machine learning models including Support Vector Machine (SVM), Gaussian Naïve Bayes (GNB), Multinomial Naïve Bayes (MNB), KNN, Logistic Regression (LR), and Random Forest (RF). The developed model is interpreted using an explainable artificial intelligence (XAI) model known as partial dependence plot.

**Results:**

A total of 137 subjects aged between 9 and 93 years were recruited for this study, 54 experienced clinical symptoms were considered positive tests, while the remaining 83 tested negative. Optimal results were obtained by combining the KNN imputation technique and three tilting features with SVM with 90.5% accuracy, 87.0% sensitivity, 92.7% specificity, 88.6% precision, 87.8% F1 score, and 95.4% ROC (receiver operating characteristics) AUC (area under curve).

**Conclusions:**

The proposed algorithm effectively classifies VVS patients with over 90% accuracy. However, the study was confined to a small sample size. More clinical datasets are required to ensure that our approach is generalizable.

## Introduction

Syncope is characterized by transient loss of consciousness resulting from global disruption in cerebral perfusion [1]. It strikes quickly followed by rapid, complete recovery. Accounting for 1% to 3% of all consultations at emergency rooms, 40% are then admitted to hospital for etiological investigations [2, 3]. While the presence of syncope may herald serious life-threatening conditions, a large number of such cases are attributed to vasovagal syncope (VVS), which is a non-life threatening condition [4, 5].

Vasovagal syncope is often triggered by specific actions, such as standing for prolonged periods, urination, or experiencing a frightening event. The underlying pathophysiology of VVS is intricate. To put it briefly, certain proactive situations can cause the vagus nerve to act abnormally, leading to a sudden drop in blood pressure (BP), decreased heart rate (HR) (bradycardia), widening of blood vessels (vasodilation), excessive sweating (diaphoresis), and various symptoms like dizziness, light-headedness, nausea, blurred vision, ultimately resulting in syncope (fainting) [6–9]. The exact reasons for this exaggerated response involve various factors, including an overactive parasympathetic response, heightened sensitivity of the blood vessels, and abnormal signalling within the autonomic nervous system (ANS) [10]. Moreover, these factors contribute to an abnormal reflex response to triggers such as orthostatic stress or emotional stimuli, leading to the characteristic symptoms and fainting episodes associated with VVS [11]. These physiological changes are a result of the ANS response and can be triggered during the head-up tilt test (HUTT).

The head-up tilt test is a diagnostic test used to identify VVS [12]. The test is considered positive if there are physiological changes such as bradycardia, cardiac pauses, and hypotension that occur alongside an exact reproduction of symptoms that the patient had previously experienced during their spontaneously occurring episodes [13]. Therefore, the key requirement for diagnosing VVS using HUTT is to provoke the unpleasant and often distressing symptoms associated with VVS. Additionally, the test is time-consuming and necessitates medical supervision, as positive results may involve prolonged periods of asystole and hypotension, making it resource-intensive in terms of requiring the presence of technicians and physicians.

The aim of the study was to investigate the significance of different physiological indicators in predicting VVS. The hypothesis was that systolic blood pressure (SBP), diastolic blood pressure (DBP), and their time- and frequency-domain variables could be more significant indicators compared to HR and heart rate variability (HRV). The research explored the predictive power of these indicators in detecting VVS. Additionally, the study utilized explainable artificial intelligence (XAI) through partial dependence plot (PDP) analysis to enhance interpretability and assess the impact of input variables on prediction outcomes. By examining multiple physiological indicators and emphasizing the practical relevance of the model, the research contributes to a more comprehensive understanding and potential practical application in VVS prediction. The main objective of the research was to develop an algorithm for categorizing VVS based on electrocardiogram (ECG) and BP signals collected during the HUTT. Therefore, this research study could potentially improve the diagnostic and treatment procedures for VVS, which would benefit both healthcare providers and patients.

This study proposes a novel algorithm for the classification of VVS patients using physiological data, including ECG and BP signals, obtained during HUTT. Our algorithm incorporates a strategic combination of techniques, including data imputation and feature selection, and employs six ML classifiers, namely, SVM, KNN, GNB, MNB, LR, and RF, to achieve a significant improvement in the accuracy of VVS classification. A unique feature of our algorithm is the integration of XAI, as demonstrated by the utilization of PDP analysis. This innovative approach not only enhances the model's performance, but also enriches its comprehensibility. As a result, healthcare providers can gain a deeper understanding of the complex relationships between ECG and BP signals from HUTT and the prediction of VVS. This facilitates the seamless implementation of our model in clinical settings and provides significant benefits to both patients and medical practitioners.

## Results

Eighty-three test negative patients, mean age 65.35 ± 19.99 years, and 54 test positive subjects, mean age 66.24 ± 20.77 years were included. The study employed two datasets, dataset one (D1) imputed using the KNN method, and dataset two (D2) using the mean method. Both imputation methods were used to produce all the results. The entire data range is displayed as a mean with a standard deviation (SD). A preliminary preview of our study was presented here [14].

### Selected features and statistical analysis of the proposed methods

The electrocardiographic and BP signals were studied in the time and frequency domains during tilt and while supine, yielding a total of 55 parameters that were utilized to the feature importance (FI) algorithm. Three identical features were chosen using the FI approach from both D1 and D2. They are all tilting features named 'CV_SBPV' (coefficient of variance of systolic BP variability)', CV_DBPV' (coefficient of variance of diastolic BP variability), ' LFnu_SBPV' (low-frequency normalized power of systolic BP variability). The feature importance values for the two imputation methods were different. Our study used several combinations of parameters for the genetic algorithm (GA) and the optimal values for D1 were crossover probability (CP) = 0.5, mutation probability (MP) = 0.1, crossover independent probability (CIP) = 0.4 and mutation independent probability (MIP) = 1. For D2, however, CP = 0.5, MP = 0.2, CIP = 0.5, and MIP = 1 was the most effective (Table 1).

**Table 1:** Selected features and GA parameters value using D1 and D2

No.	CP	MP	CIP	MIP	Tournament size	Scores	Features
Value of selected features of GA by D1							
1	0.5	0.1	0.4	0.05	1	93.2	'CV_HR_S', 'SD_SBP_S', 'RMSRV_SBP_S', 'CV_DBP_S', 'CV_HR_T', 'Lfnu_SBP_T', 'SD_DBP_T', 'CV_DBP_T'
2	0.5	0.2	0.4	0.05	1	92.79	Hfnu_RRI_S', 'Hfnu_SBP_S’, ‘SBP_T', 'CV_SBP_T', 'CV_DBP_T'
3	0.5	0.2	0.5	0.05	1	93.39	Age', 'ARV_SBP_S', 'SDRV_SBP_S', 'Hfnu_SBP_S', 'Lfnu_SBP_S', 'CV_DBP_S', 'CV_SBP_T', 'Lfnu_DBP_T'
4	0.5	0.2	0.5	0.04	1	93.1	'Hfnu_RRI_S', 'CV_SBP_S', 'LFHF_RRI_T', 'CV_SBP_T', 'SDRV_SBP_T', 'Lfnu_SBP_T', 'CV_DBP_T'
5	0.5	0.2	0.5	0.04	3	95.0	'SD_SBP_S', 'DBP_S', 'CV_DBP_S', 'RMSRV_DBP_S', 'SD_HR_T', 'SBP_T', 'CV_SBP_T', 'Lfnu_SBP_T', 'SD_DBP_T', 'CV_DBP_T'
Value of selected features of GA by D2							
1	0.5	0.1	0.4	0.05	1	94.3	'DBP_S', 'Hfnu_DBP_S', 'SBP_T', 'CV_SBP_T', 'SDRV_SBP_T', 'Lfnu_SBP_T', 'LFHF_SBP_T', 'CV_DBP_T', 'Lfnu_DBP_T'
2	0.5	0.2	0.4	0.05	1	93.3	'Age', 'Hfnu_SBP_S', 'Lfnu_DBP_S', 'CV_HR_T', 'SDRV_HR_T', 'CV_SBP_T', 'SDRV_SBP_T', 'SD_DBP_T', 'Lfnu_DBP_T'
3	**0.5**	**0.2**	**0.5**	**0.05**	**1**	**93.2**	**'Hfnu_RRI_S', 'SD_SBP_S', 'DBP_Supine', 'SDRV_DBP_S', 'SD_HR_T', 'SDRV_HR_T', 'SBP_T', 'ARV_SBP_T', 'CV_DBP_T', 'Lfnu_DBP_T'**
4	0.5	0.2	0.5	0.04	1	93.1	'SD_SBP_S', 'SDRV_SBP_S', 'CV_SBP_T', 'ARV_SBP_T', 'Lfnu_SBP_T', 'SD_DBP_T', 'CV_DBP_T', 'Hfnu_DBP_T'
5	0.5	0.2	0.5	0.04	3	95.8	'Age', 'DBP_S', 'CV_DBP_S', 'LFHF_RRI_T', 'SBP_T', 'CV_SBP_T', 'Lfnu_SBP_T', 'SD_DBP_T', 'CV_DBP_T'

Bold values indicate the best performance of the model

D1, K-nearest neighbors (KNN) imputation data; D2, mean imputation data; S, supine; T, 70 degree tilting; CP, crossover probability; MP, mutation probability; CIP, crossover independent probability; MIP, mutation independent probability = MIP; HR, heart rate; HRV, HR variability; SBP, systolic BP; DBP, diastolic BP; SBPV, SBP variability, DBPV, DBP variability; CV, coefficient of variance; ARV, average real variability; RMSRV, root mean square of real variability; SDRV, standard deviation of real variability; HFnu, normalized high-frequency power; LFnu, normalized low-frequency power; LFHF, normalized ratio of HF to LF

The study maintained a constant population size of 100 and generation number of 10 for both D1 and D2. Our algorithm would probably find local optimum conditions when the population size was too small since the population would lose its diversity. Furthermore, the replacement stage involves some challenges. The study had to exclude the impractical solutions since the population size was too small. The general rule is that the larger the population, the better, but the study frequently must make concessions to accomplish our goal in a fair amount of time. The ideal values for many parameters must be conjectured by the study when creating a GA. No satisfactory response exists. In general, our investigation was conducted through trial and error. As per both D1 and D2, the model defined three identical important features. The genetic algorithm method, applied to D1 and D2 data, has successfully identified two set of relevant features (Table 1). The parameters, chosen via the recursive feature elimination (RFE) method using both D1 and D2 data, unveiled features like 'DBP' and 'CV_DBPV' in the tilting position, along with 'SBP' in the supine position.

For the application of the Mann–Whitney *U* Test (MWUT), the parameter should be ordinal or continuous but not necessarily normally distributed, and the two groups must be distinct [15]. These are the sole prerequisites. In our case, when comparing the supine rested data of test-positive and test-negative subjects (Table 2), no statistically significant difference was observed. The tabulated results affirm the absence of noteworthy distinctions between the supine rested findings of the test positive and test negative groups.

**Table 2:** The Mann–Whitney U test (MWUT) results of all the selected features of FI, GA, and RFE

Features	Supine position			HUTT position		
	NEG	POS	*P* value	NEG	POS	*P* value
CV_SBPV	6.17 ± 3.01	6.08 ± 3.66	0.50	8.35 ± 3.12	18.82 ± 7.11	< 0.001
CV_DBPV	7.87 ± 3.80	7.20 ± 3.25	0.25	9.81 ± 5.72	18.96 ± 6.83	< 0.001
LFnu_SBPV	36.79 ± 12.26	33.14 ± 12.67	0.09	49.09 ± 16.98	34.41 ± 13.59	< 0.001
CV_HRV	12.55 ± 11.44	12.19 ± 8.91	0.62	10.54 ± 7.30	15.34 ± 8.19	< 0.001
Hfnu_HRV	53.83 ± 18.46	56.27 ± 20.79	0.38	47.55 ± 21.74	52.11 ± 19.57	0.32
SD_SBPV	7.81 ± 3.27	6.59 ± 3.72	0.19	10.42 ± 4.23	18.34 ± 6.27	< 0.001
RMSRV_SBPV	3.64 ± 3.31	3.67 ± 2.72	0.69	3.72 ± 3.26	4.28 ± 3.19	0.42
SD_DBPV	5.60 ± 2.25	5.04 ± 1.98	0.10	7.49 ± 2.87	12.45 ± 4.26	< 0.001
ARV_SBPV	2.18 ± 1.96	2.19 ± 1.76	0.73	2.55 ± 2.05	2.61 ± 1.43	0.51
Lfnu_DBPV	42.45 ± 14.61	39.61 ± 13.84	0.28	50.93 ± 17.84	37.84 ± 12.60	< 0.001
SDRV_DBPV	3.61 ± 2.66	3.83 ± 2.74	0.37	3.39 ± 2.35	3.50 ± 1.98	0.08
SD_HRV	9.34 ± 9.68	8.86 ± 6.49	0.51	9.67 ± 7.41	13.53 ± 8.19	0.01
SDRV_HRV	11.58 ± 13.91	11.72 ± 10.12	0.37	9.48 ± 11.82	11.16 ± 13.16	0.089
SBP	118.15 ± 11.82	112.80 ± 18.04	0.08	125.06 ± 15.03	100.29 ± 17.73	0.01
DBP	73.43 ± 9.36	72.09 ± 9.75	0.35	80.33 ± 12.50	67.03 ± 9.61	0.01

HUTT, head-up tilt test, D1, K-nearest neighbors (KNN) imputation dataset; D2, mean imputation dataset; NEG, test negative subject; POS, test positive subjects; p < 0.01 is statistically significant and p < 0.001 is the most statistically significant values, HR, heart rate; SBP, systolic BP; DBP, diastolic BP; DBPV, DBP variability; CV, coefficient of variance; ARV, average real variability; HRV, HR variability; RMSRV, root mean square of real variability; SBPV, SBP variability; SDRV, standard deviation of real variability; HFnu, normalized high-frequency power; and LF; LFnu, normalized low frequency power; LFHF, normalized ratio of HF

### The proposed classifiers

The study utilized six distinct ML models namely SVM, KNN, GNB, MNB, LR, and RF for binary classification. Hyperparameter tuning was systematically performed for each model via grid search. This method involved the exploration of parameter combinations to optimize the model configuration, thereby improving predictive accuracy and generalization capabilities. This meticulous process enabled the fine-tuning of hyperparameters for every model, resulting in dependable and resilient outcomes.

Due to random data division for ML model evaluation, imputed data may exist in the test subset, using mean and KNN imputation. With 80% for training and 20% for testing, some imputed instances could be in the test set. While prioritizing unimputed data in testing, the stochastic process can lead to imputed points in the test set. This approach balances rigorous assessment with practicality, providing insights into algorithm performance with authentic and imputed data.

By assessing a ML model's performance on novel data with less bias, the approach assesses its generalizability. The fivefold cross-validation approach verifies the outcomes after the model has been developed using train-test-split. The best results were obtained when the KNN imputation method and three tilting features—CV_SBPV, CV_DBPV, and LFnu_SBPV were combined with SVM. For the support vector machine model, the ideal set of hyperparameters was c = 1, gamma = 0.01, and kernel = RBF. The best result was obtained with 90.5% accuracy, 87.0% sensitivity, 92.7% specificity, 88.6% precision, 87.8% F1 score and 95.4% ROC (AUC) (Table 3). In the proposed optimal SVM model, TP (true positive), TN (true negative), FP (false positive), and FN (false negative) are, 47, 77, 6, and 7, respectively.

**Table 3:** The outcome summary of the proposed models by D1 and D2

Classifiers	Accuracy	Sensitivity	Specificity	Precision	F1 score	ROC_AUC
Classification results of FI using D1						
SVM	90.5	87.0	92.8	88.7	87.9	95.4
KNN	87.5	75.9	95.2	91.1	82.8	95.7
GNB	86.1	75.9	92.8	87.2	81.2	92.9
MNB	86.9	75.9	93.9	89.1	82.0	91.7
LR	86.1	75.9	92.8	87.2	81.2	93.6
RF	87.6	79.6	92.8	87.8	83.5	94.9
Classification results of FI using D2						
SVM	89.1	83.3	92.8	88.2	85.7	93.2
KNN	88.3	83.3	91.6	86.5	84.9	96.3
GNB	83.9	66.7	95.2	90.0	76.6	91.7
MNB	87.6	75.9	95.2	91.1	82.8	91.7
LR	85.4	74.1	92.8	86.9	80.0	93.1
RF	86.1	75.9	92.8	87.2	81.2	94.9
Classification results of GA using D1						
SVM	89.8	81.5	95.2	91.7	86.3	94.9
KNN	84.7	75.9	90.4	83.7	79.6	95.5
GNB	84.7	77.8	89.2	82.4	80.0	92.8
MNB	86.1	79.6	90.4	84.3	81.9	92.6
LR	86.1	81.5	89.2	83.0	82.2	93.9
RF	82.5	75.9	86.7	78.8	77.4	97.3
Classification results of GA using D2						
SVM	86.1	87.0	85.5	79.7	83.2	99.7
KNN	76.6	61.1	86.7	75.0	67.3	90.9
GNB	78.1	72.2	81.9	72.2	72.2	89.3
MNB	79.6	72.2	84.3	75.0	73.6	87.8
LR	89.8	87.0	91.6	87.0	87.0	98.7
RF	83.9	74.1	90.4	83.3	78.4	97.3
Classification results of RFE using D1						
SVM	83.2	75.9	87.9	80.4	78.1	88.5
KNN	77.4	68.5	83.1	72.5	70.5	90.8
GNB	79.6	64.8	89.2	79.5	71.4	87.7
MNB	84.7	81.5	86.7	80.0	80.7	88.3
LR	81.8	70.4	89.2	80.9	75.2	88.5
RF	83.2	74.1	89.2	81.6	77.7	94.0
Classification results of RFE using D2						
SVM	83.2	75.9	87.9	80.4	78.1	88.5
KNN	77.4	68.5	83.1	72.5	70.5	90.8
GNB	79.6	64.8	89.2	79.5	71.4	87.7
MNB	84.7	81.5	86.7	80.0	80.7	88.3
LR	81.8	70.4	89.2	80.9	75.2	88.5
RF	83.2	74.1	89.2	81.6	77.7	94.0

FI, feature importance; GA, genetic algorithm; RFE, recursive feature elimination; D1, K-nearest neighbors (KNN) imputation dataset; D2, mean imputation dataset; SVM, support vector machine; GNB, Gaussian naïve Bayes; MNB, multinomial naïve Bayes; LR, logistic regression; RF, random forest

The three selected tilting features, named 'CV_SBPV', 'CV_DBPV' and 'LFnu_SBPV' indeed exhibit a strong relevance to the pathology of VVS, particularly during the HUTT. These features provide valuable insights into the physiological dynamics that underlie VVS and contribute to its classification. 'CV_SBPV' and 'CV_DBPV', which quantify changes in BP during HUTT, align with a critical aspect of VVS pathology. A decrease in BP during HUTT is a well-documented phenomenon in individuals with VVS. This drop in BP can be a primary contributor to VVS episodes. 'LFnu_SBPV', measuring the balance between the sympathetic nervous system (SNS) and parasympathetic nervous system (PNS), further highlights the intricate autonomic involvement in VVS. The role of the SNS and the PNS in regulating HR and BP is pivotal. The observed decrease in SNS activity during HUTT aligns with the pathophysiology of VVS. Such changes can lead to inadequate cardiovascular responses potentially triggering a VVS episode.

The robustness of (CV_SBPV and CV_DBPV) becomes evident, as they provide a normalized assessment of variability through the SD-to-mean ratio. This normalization effectively accounts for mean value differences among individuals—a crucial consideration absent in the standalone SD. The latter’s limitation in capturing the full extent of variability in cross-individual comparisons underscores the significance of (CV_SBPV and CV_DBPV). Moreover, (CV_SBPV and CV_DBPV) outshine ARV due to their dual capability of gauging relative variability and embracing the average value. In contrast, ARV's sole focus on absolute variations between consecutive data points might overlook the intricate autonomic dynamics integral to VVS events. The application of (CV_SBPV and CV_DBPV) in beat-to-beat data analysis plays a pivotal role, capturing dynamic fluctuations in SBP, DBP, and HR over temporal intervals, revealing autonomic regulatory patterns that enhance their diagnostic significance. Meanwhile, LFnu_SBPV offers a unique edge over RMSRV by unveiling the equilibrium between the SNS and PNS. While RMSRV captures sequential differences, LFnu_SBPV delves into the autonomic balance, a vital determinant in syncope assessment. Furthermore, comparing (CV_SBPV and CV_DBPV) against SDRV, the former's normalized measure of variability stands out, effectively highlighting subject distinctions. On the contrary, SDRV's assessment of sequential differences without normalization for mean values might hinder its ability to differentiate subjects optimally. Therefore, the comprehensive and normalized nature of (CV_SBPV, CV_DBPV, and LFnu_SBPV) prevails in contrast to SD, ARV, RMSRV, and SDRV.

These three selected features reveal intricate physiological insights and excel in beat-to-beat analysis, enhancing VVS episode understanding and classification. These quantitative foundations enable a more precise diagnostic approach, potentially improving patient care. Their significance in VVS classification during HUTT underscores their clinical relevance.

### Receiver operating characteristics curve

**Fig. 1:**
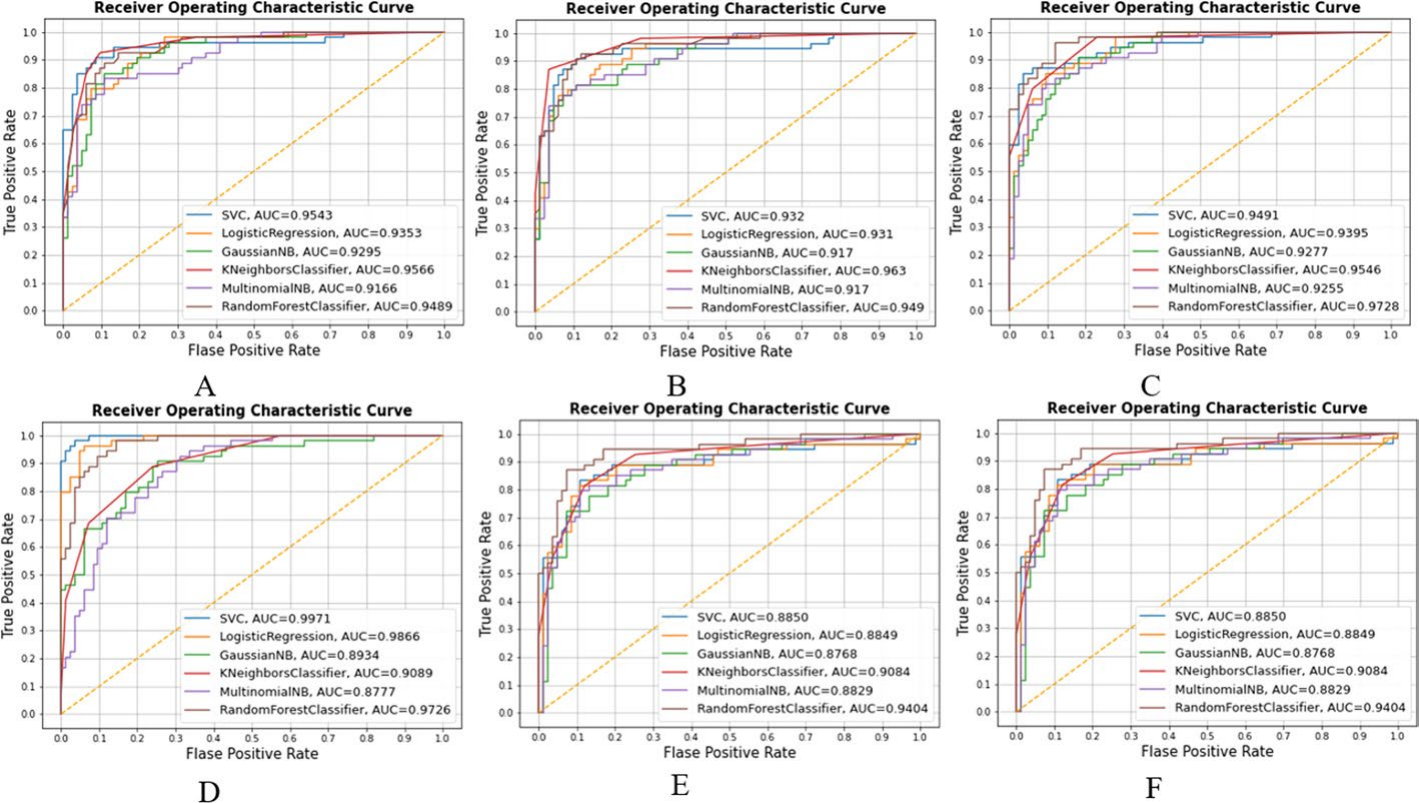
Receiver operating characteristics curve by D1 and D2. (Here **A** KNN imputation data + FI selected features; **B** mean imputation data + FI selected features; **C** KNN imputation data + GA selected features; **D** mean imputation data + GA selected features; **E** KNN imputation data + RFE selected features; **F** mean imputation data + RFE selected features.)

The receiver operating characteristics curve is a technique for evaluating the ability to distinguish between test positive and negative subjects at various threshold levels. A good model will have an area under curve (AUC) near to 1, which denotes a high degree of separability. 90.5% accuracy and 95.4% ROC were found to be ideal in the study employing D1 and FI chosen features (Table 3). Employing D2 with GA-selected features yielded the maximum 99.7% ROC (Fig. 1A), although accuracy was 86.1% (Fig. 1D). As a result, it is conceivable for an elevated AUC classifier to fail to perform below a lower AUC classifier in a specific area of the ROC space.

### Violin plot

**Fig. 2:**
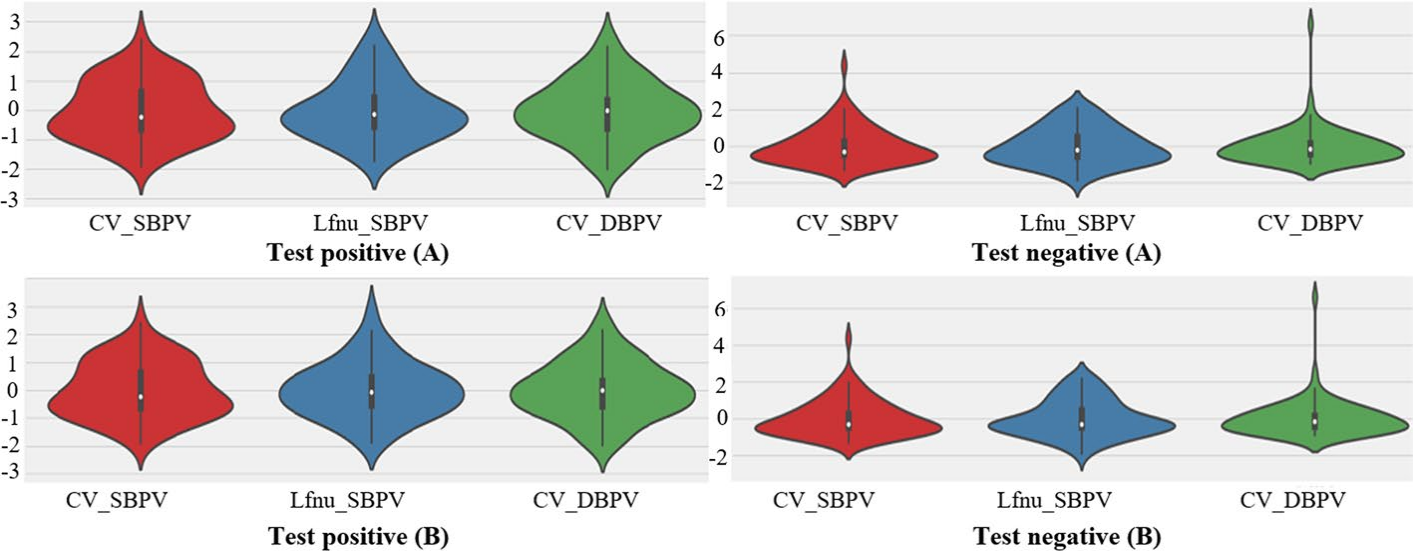
Violin plot of effects of test positive and negative subjects on tilting position variables of CV_SBPV (coefficient of variance of systolic BP variability), CV_DBPV (coefficient of variance of diastolic BP variability), and LFnu_SBPV (low-frequency normalized power of systolic BP variability). The estimated likelihood density of the data at test positive and test negative individuals is also displayed on the violin plot, providing a more thorough comprehension of the distribution of the values. Here **A** KNN imputation data + FI selected features; **B**  mean imputation data + FI selected features

The significance of the violin plot in VVS classification lies in its ability to visually represent the data’s probability density, even with non-normally distributed data. Additionally, it enables effective comparison of variable distributions between test-positive and test-negative subjects, aiding in the identification of crucial differences for developing a reliable syncope classification model. Figure 2 represents the violin plot.

### The outcome of partial dependence plot

**Fig. 3:**
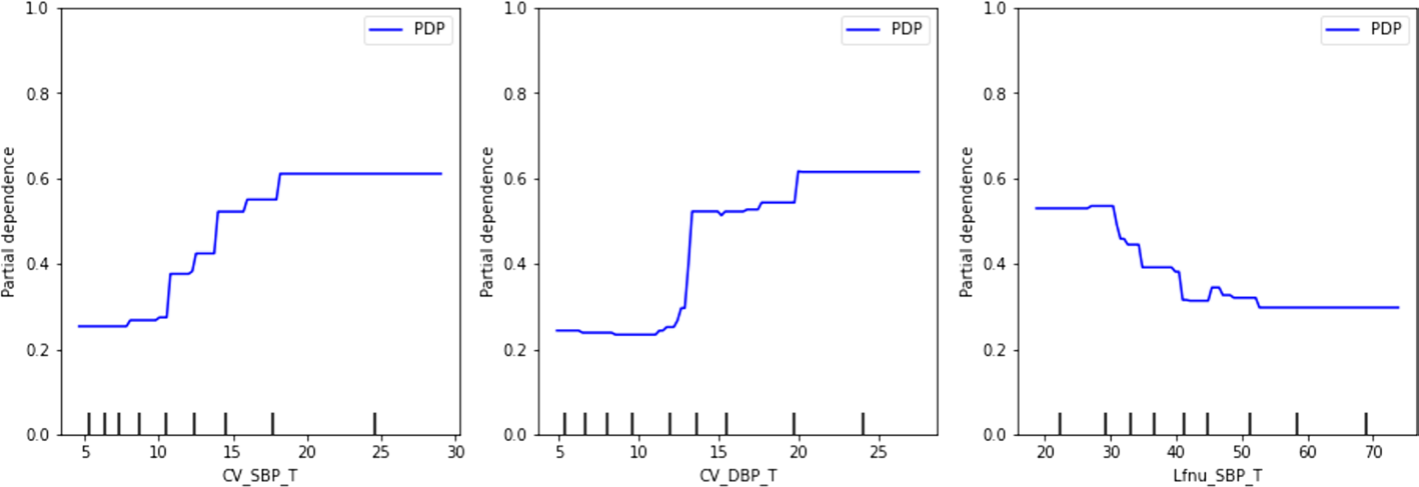
The partial dependence plot by D1(KNN imputation) data. According to feature importance, three tilting features named "CV_SBPV" (coefficient of variance of systolic BP variability), "CV_DBPV" (coefficient of variance of diastolic BP variability), and "LFnu_SBPV" (Low-frequency normalized power of systolic BP variability) were chosen, and this feature combination produced the best accuracy. Here, x axis CV_SBPV_T, CV_DBPV_T and Lfnu_SBPV_T represent the fixed values of target features and y axis represents the probability of predicted risk of a positive test. The subjects were more likely to test positive with higher CV_SBPV_T and CV_DBPV_T, while positive tests had lower Lfnu_SBPV_T

In the partial dependence plot, the expected risk for the initial data, was displayed as a blue curve. PDP values exceeding 0.5 indicated a higher probability of a positive test. Higher CV_SBPV values were associated with positive tests, while lower CV_DBPV values were associated with negative tests. LFnu_SBPV values were conversely lower in those with positive tests (Fig. 3).

## Discussion

The significance of predicting VVS occurrence lies in the development of proactive prevention techniques and targeted clinical interventions. By creating an algorithm to classify individuals with VVS based on physiological data, including BP and ECG, acquired during the HUTT features which play a vital role in the classification of VVS test positive and VVS test negative subjects, crucial information about the physiological differences between these two groups has been revealed. The ML models developed are able to classify VVS with high accuracy (over 90%). Previous studies have mainly applied resting state features. For instance, the study by Kostoglou et al. achieved a predictive performance metric of *ρ* = 0.952 in forecasting the time to syncope occurrence (TSO) in VVS patients using only resting state features [16]. Furthermore, Kostoglou et al. focused exclusively on ECG signals, overlooking other vital physiological indicators like HR, which could limit the comprehensiveness of their predictive performance. Another noteworthy difference is that Kostoglou et al. primarily utilized a single ML algorithm (RF), potentially missing the opportunity to explore alternative algorithms that might achieve even better predictive performance. Evangelia Myrovali et al. were able to predict syncope outcomes from resting state clinical data with an accuracy of 89.7% [17]. The two studies mentioned above had utilized relatively small datasets of 71 and 26 patients, respectively, raising concerns about their applicability to larger and more diverse patient populations. Additionally, their primary goal of predicting syncope outcomes may hinder the direct application of their findings to patient classification, which is a crucial aspect of VVS diagnosis and management.

In contrast, our study overcame these limitations with a more extensive dataset of 137 patients, greatly improving the potential for generalization to a broader VVS patient population compared to the previous studies. Furthermore, we employ a comprehensive approach by incorporating both ECG and BP signals, capturing data from both supine and tilting positions during HUTT, leading to a classification accuracy of 90.5%. Additionally, we addressed the limitation of using a single ML algorithm by evaluating a wide range of methods, including KNN, SVM, GNB, MNB, LR, and RF, ensuring a more robust exploration of classification techniques. Our study stands out for its comprehensive approach to VVS patient classification, incorporating tilting features in the feature selection process, and offering a versatile tool directly applicable to clinical practice for diagnosis and management.

Turning to R. Couceiro et al., their focus on predicting Neurally Mediated Syncope (NMS) in real-time, with high sensitivity and specificity, offers promise for continuous monitoring and wearable systems [3]. However, their concentration on real-time prediction may not align directly with the need for patient classification, which is essential for diagnosis and management in a clinical setting. Our study distinguishes itself by centering on the classification of VVS patients using data collected during the HUTT, providing practical clinical applications. While R. Couceiro et al. excel in the real-time prediction of NMS episodes, our research broadens its scope by categorizing VVS patients based on their physiological responses during HUTT. Therefore, our study surpasses the limitations of previous research through a more extensive dataset, consideration of multiple physiological signals, and a diverse range of ML algorithms. It offers a comprehensive approach to VVS patient classification and provides clear potential for practical clinical applications in the diagnosis and management of VVS.

### Performance evaluation with state-of-the-artwork

Ciliberti, M. A. P., et al. [18] investigated the potential of resting HRV spectral components to predict VVS in patients referred for HUTT due to unexplained syncope. Their initial findings indicate that HRV analysis could serve as a predictive tool for identifying individuals at risk of VVS. However, it is crucial to emphasize that further research is essential to validate and build upon these initial results, enhancing our comprehension of this predictive mechanism and its clinical significance. The primary limitation of the study is the relatively small sample size, which included only 26 patients. M. Kwok et al. 2020 had investigated ML models to determine whether syncope occurred in persons aged 50 years and older [19] and M. Carmody et al. 2020, examined younger patients with a mean age of 25 ± 9 years [20]. We have included age as one of the syncope classification variables which was selected by GA. However, the combination of age and related features (Table 1) were unable to yield results with a better accuracy level. Therefore, our study did not suggest age as an indicator of syncope. He, Z., et al. [21] conducted a study where they recorded various beat-to-beat physiological parameters and developed ML algorithms to achieve early prediction of HUTT outcomes. Their approach successfully reduced the original 35-min tilting duration to just 13 min. However, the study lacks details regarding the specific methods they used to shorten the prediction time during HUTT and how early in the process they were able to predict the occurrence of VVS. This lack of information makes it challenging to assess the full implications and applications of their findings. S. Hussain et al. 2022 had exhibited higher accuracy (98.9%), sensitivity (97.6%), specificity (92.7%), and precision (92.2%), as well as an F1 score (94.9%) and ROC (AUC) (98.3%) than ours. The balance of their dataset is, however, questionable with only 96 test-positive patients and 591 test-negative ones [22]. Standard classifiers frequently disregard the little classes in these situations because they are too overwhelmed with the large classes. In that case, it would not be a true reflection of the overall picture. Nevertheless, they employed the synthetic minority oversampling technique (SMOTE), which is an effective oversampling methodology. However, SMOTE has three drawbacks: oversampling unhelpful sample sizes, noisy samples are oversampled, and challenges in estimating the number of nearest neighbors with significant blindness in the selection of nearest neighbors for the synthetic samples [23]. Alternative strategies such as Borderline-SMOTE, RCSMOTE, K-means SMOTE to address these shortcomings had not been utilized [22]. Previously, S. Hussain et al. 2021 published a SVM-based classification which was restricted to using SVMs for classification and did not explore different supervised ML algorithms that would have produced an improved classification [24]. Unexpectedly, neither HR nor HRV indices were chosen from our feature selection. There is a considerable difference between this and other studies. In Table 4, none of the studies provided explanations within their machine learning models regarding how the features influenced their performance. Notably, only our study had incorporated an explainable artificial intelligence (XAI) model known as PDP. This disparity represents a significant difference between our research and the others, as it enhances the transparency and interpretability of our model by illustrating how specific features impact the overall performance. This detailed insight into feature importance sets our study apart, providing a deeper understanding of the underlying mechanisms driving the classification of VVS.

**Table 4:** Performance evaluation with state-of-the-artwork

Authors	Results (%)	Selected features	Sample size
1. Ciliberti, M. A. P., et al. 2018 [18]	Sensitivity: 87.5 Specificity: 72.2 PPV: 75.0 NPV: 89.0	HRV, VLF, LF, HF, LF/HF ratio	26 (all pos)
2. M. Kwok et al. 2020 [19]	Sensitivity: 82.6 Specificity: 76.8 Accuracy: 78.9	HR, CO, SV, TPR, SBP, DBP, HRV, BPV	128 (46 pos and 82 con)
3. Carmody et al. 2020 [20]	Sensitivity: 58.8 Specificity: 63.3 Accuracy: 80.2 ROC(AUC):83.2	SBP-5 s%, SBP-20 s%, HR-%I, CO- %I, SV-B2SS	101 (34 pos, 30 neg and 37 con)
4. He, Z., et al. 2021 [21]	Sensitivity: 86.0 Specificity: 82.0 ROC(AUC): 94.0	HR, RRI, SBP, DBP, MBP, LVET, TPR, CO, SV	203 (128 pos, 75 neg)
5. S. Hussain et al. 2022 [22]	Sensitivity: 97.6 Specificity: 92.7 Precision: 92.2 F1 score: 94.9 Accuracy: 98.9 ROC(AUC):98.3	HR, SV, CO, CI, SI, RRI, TPR, TPRI, DBP, MBP, SBP	687 (96 pos and 591 neg)
6. Proposed	Sensitivity: 87.0 Specificity: 92.7 Precision: 88.6 F1 score: 87.8 Accuracy: 90.5 ROC(AUC):95.4	CV_SBPV, CV_DBPV, LFnu_SBPV	137 (54 pos and 83 neg)

neg, test negative; con, control (healthy subject); POS, test positive; PPS, psychogenic pseudosyncope; PPV, positive predictive value; NPV, negative predictive value; RRI, R–R interval; HR, heart rate; HRV, hear rate variability; MBP, mean blood pressure; SV, stroke volume; CO, cardiac output; TPR, total peripheral resistance; LVET, left ventricular ejection time; SBPV, SBP variability; DBPV, DBP variability; SI, stroke Index; TPRI, total peripheral resistance index; (SBP-5 s%, SBP-20 s%), the percentage change in SBP from baseline at 5 s and 20 s post-stand; (HR-%I, CO- %I), the percentage increase from baseline of heart rate and CO and; (SV-B2SS), the percentage difference in SV from baseline to steady state; CV, coefficient of variance; LFnu, low-frequency normalized unit; HF, high frequency; VLF, very high frequency

Moreover, our study demonstrates the superior performance of our proposed algorithm in accurately identifying positive cases of VVS, with higher levels of accuracy and sensitivity compared to previous studies, except for the study conducted by S. Hussain et al. [22] (Table 4). The key distinction of our approach is its reliance on just three parameters, setting it apart from others. This simplification of the classification process improves practicality and reduces computational complexity. The study introduces a new algorithm for syncope detection that utilizes only three parameters out of the 55 initially extracted features. This streamlined approach eases integration into existing diagnostic systems and clinical workflows. Additionally, the reduced computational complexity of the algorithm enables faster processing times, making it well-suited for real-time applications. Importantly, the simplified nature of the algorithm enhances interpretability, a crucial factor in the medical field. This allows clinicians to trust and validate the results obtained from the algorithm.

Previous studies have shown that conventional algorithms often necessitate a larger set of features, typically ranging from five to ten, to effectively classify patients with syncope (Table 4). However, our study highlights the significance of specific parameters—CV_SBPV, CV_DBPV, and LFnu_SBPV—which contribute to improved performance of our algorithm. This novel approach offers insights into the physiological factors associated with syncope and suggests potential biomarkers for future investigations. The high accuracy and sensitivity demonstrated by our algorithm underscore its practical potential for clinical implementation. By providing healthcare professionals with an efficient and accurate syncope diagnosis, it facilitates timely interventions and improves patient outcomes.

## Importance in clinical application

Our research significantly advances the approach to diagnosing and managing VVS, providing a more precise and efficient method for the diagnosis of VVS. By utilizing physiological data, specifically BP and ECG collected during the HUTT, we can accurately categorize individuals with VVS. This categorization allows for the development of personalized treatment plans tailored to each patient's unique physiological characteristics. Furthermore, our study contributes to a deeper understanding of the underlying pathophysiology of VVS, potentially guiding the development of innovative and more efficient treatment approaches. A distinctive aspect of our algorithm involves the incorporation of XAI, illustrated by the application of PDP analysis. This, in turn, has the potential to lead to improved clinical interventions and better outcomes for individuals with VVS.

## Limitations

Some individuals with VVS could have had a false negative test, which would not be considered in this classification model. Since all individuals had HUTT because of a clinical suspicion of VVS, those who tested negative would still have suspected syncopal symptoms which could not be attributed to VVS. Nevertheless, this would have been a clinically relevant observation, as using this classifier, unpleasant and distressing symptoms associated with HUTT could potentially be avoided. There could be future utility in differentiating those with non-life threatening VVS from those individuals with more serious causes of syncope from which sudden cardiac death may occur. As the clinical approach towards investigation of syncope would be to first rule out life-threatening causes, additional evaluation will be required before the role of such an approach could be appropriately determined. Moreover, a small sample size was used in the current research study. More clinical datasets are required for the robustness and generalizability of our method.

However, clear clinical and economic benefits exist for this potential approach since the extensive investigative approach currently employed in order to not to miss life-threatening causes, is resource-intensive, and in need of refinement [25]. Only ECG and BP signals were examined for the investigation. Patients with VVS could also be categorized with the aid of the impedance cardiography (ICG) signal [26]. It is a safe technology that assesses the total electrical conductivity of the thorax and changes in that conductivity over time to process constantly a few cardiodynamic parameters, including stroke volume, cardiac input, cardiac output, ventricular ejection time, and pre-ejection interval. It is utilized to find changes in impedance brought on by a high-frequency, low-amplitude current running through the thorax between two extra pairs of electrodes outside the measured section.

## Conclusion and future work

The significant synergy among three key tilting features—'CV_SBPV', 'CV_DBPV', and 'Lfnu_SBPV'—identified through the FI method along with D1, yielded a 90.5% accuracy when employed with the SVM model. This substantial boost in accuracy underscores the SVM's dominance over alternate classification techniques within our study. Our experimental outcomes considerably fortify the credibility of our methodology, streamlining the proactive and precise categorization of individuals exhibiting positive results in the HUTT. This potential holds considerable promise for averting of VVS through timely intervention. An intriguing facet of our investigation delves into the illumination of PDP, providing valuable insights into how specific traits influence predictions. The robust accuracy attained through the amalgamation of SVM-specific features reinforces the potential of harnessing this methodology to amplify the precision and efficacy of VVS evaluation. Further evaluation of the classification method is required using larger datasets which includes potential safety concerns to avoid missing life-threatening causes of VVS, but the potential exists for refinement of current investigative strategies for the evaluation of VVS using SVM.

Within the framework of our research, predicting VVS incidence holds paramount importance as it underpins the development of effective preventive measures and clinical interventions. In the future stages of our model's development, our focus will shift toward a more detailed analysis of physiological changes that occur in the lead-up to and during syncope incidents within the tilting window. This research direction is expected to provide invaluable insights into our model's predictive capabilities. These insights will serve as a foundation for advancing our understanding of syncope, ultimately playing a pivotal role in the formulation of early intervention and prevention strategies. Our ultimate goal is to enhance patient care and safety through these advancements, and we will delve into these aspects as part of our future work.

## Methods

### System overview

**Fig. 4:**
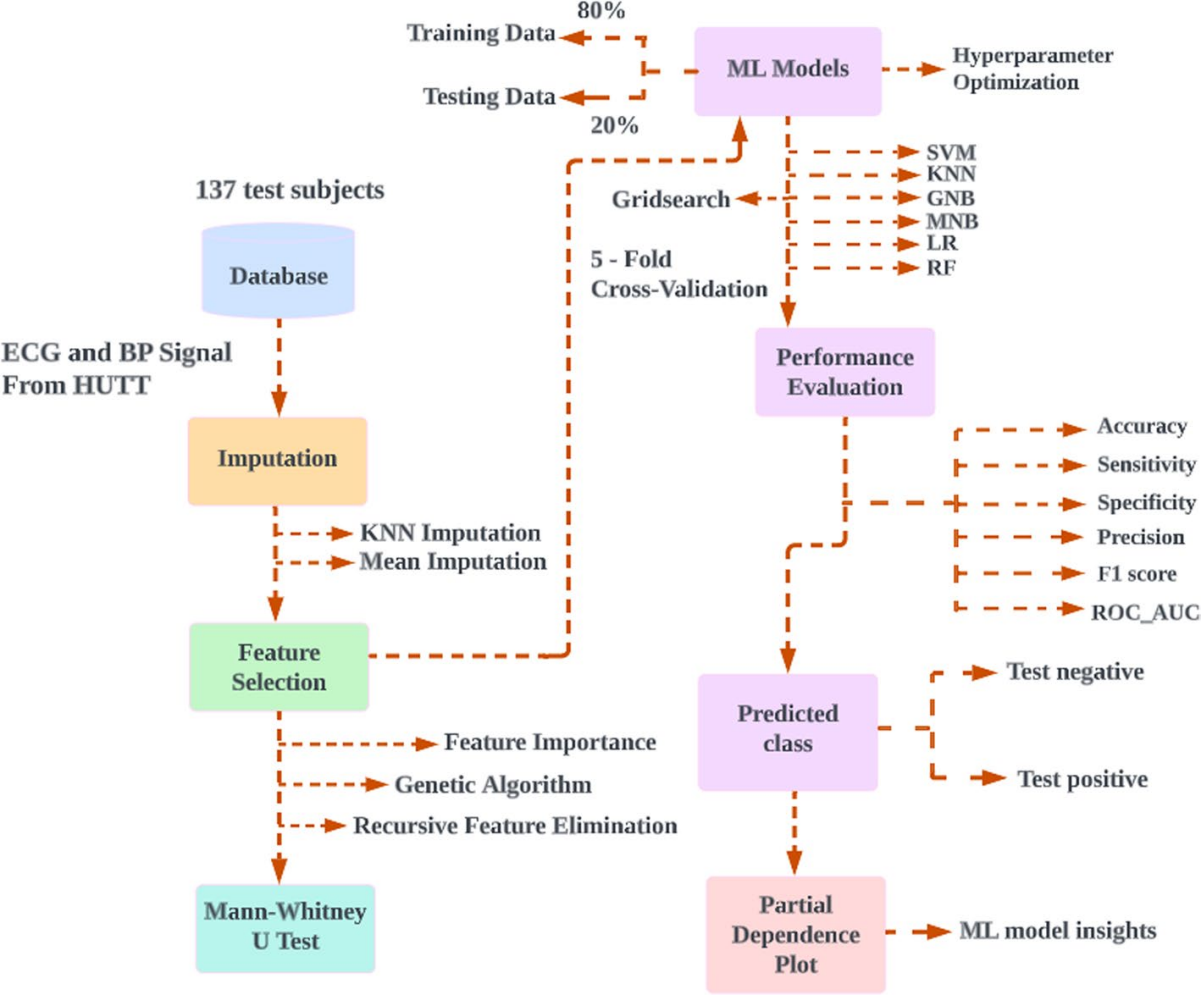
The proposed model for classification of vasovagal syncope. A flow diagram showing the proposed model for classification of vasovagal syncope. Features ECG and blood pressure signals from 137 HUTT were first extracted then imputed. Selected features identified using feature selection methods and non-parametric probability testing were performed in order to compare the statistical differences between two groups then cross validated and 80% of the data used as training set for machine learning, with the remaining 20% as testing set. The model performance was evaluated, then prediction classification and partial dependence plot applied. HUTT, head-up tilt test; ML, machine learning; SVM, support vector machine; K-nearest neighbors (KNN) imputation dataset; GNB, Gaussian naïve Bayes; MNB, multinomial naïve Bayes; LR, logistic regression; RF, random forest

The proposed method was developed with a laptop equipped with Intel (R) Core (TM) i7-6600U 2.60 GHz CPUs and 8 GB of RAM. The python packages that were used included numpy, pandas, scikit-learn, matplotlib, and seaborn. Data collection, experimental setup, data preprocessing on physiological signals, selection of features, missing value imputation, statistical analysis, ML classifiers, and PDP is all demonstrated in this section. Figure 4 represents the proposed model for classification of VVS.

### Data collection

Data collection was conducted at the Cardiorespiratory Laboratories, University of Malaya Medical Centre (UMMC). Patients were referred for HUTT as an investigation for the symptom of syncope or near syncope. All patients were provided information about the test and informed consent obtained prior to the test. Both the Universiti Tunku Abdul Rahman (UTAR) Scientific and Ethical Review Committee (U/SERC/218/2020) and the UMMC Medical Research Ethics Committee (MREC ID NO: 2,020,913–9066) provided their approval for this cross-sectional study.

### Experimental setup

The head-up tilt test was meticulously conducted, employing specialized non-invasive monitoring equipment furnished by CNSystem, with a central focus on the Task Force™ Monitor (CNSystems, Graz, Austria). This sophisticated monitoring system was purpose-built to facilitate comprehensive hemodynamic measurements, enabling seamless real-time data acquisition and subsequent analysis [27]. Core bio signals, comprising ECG and BP were meticulously recorded on a beat-to-beat basis. It is worth mentioning that the Task Force Monitor was carefully outfitted with a high-resolution two-channel ECG module, proficiently capturing data at a frequency of 1000 Hz. Additionally, a BP module, operating at 100 Hz, further augmented the system's capabilities. Subjects underwent meticulous preparation, entailing the precise attachment of electrodes and sensors to capture pivotal physiological parameters such as BP and HR. These well-prepared subjects were subsequently positioned on an integrated tilt table, thoughtfully designed to enable controlled angle adjustments. The tilt table served as a dynamic platform, allowing subjects to undergo a gradual transition from a supine position to a predetermined 70-degree angle. The protocol HUTT was carried out in a tranquil and carefully managed setting designed to minimize external factors. The substantive phase of the test commenced after a designated 10-min period of supine rest. The substantive phase of the test commenced after a designated 10-min period of supine rest. Subsequently, the tilt table was artfully maneuvered to achieve a consistent 70-degree angle, sustained total a period of 35 min [28]. Notably, a pharmacological stimulus was introduced after the initial 20 min of tilting, which involved administering 400 µg of glyceryl trinitrate (GTN) sublingually. This deliberate measure aimed to evoke specific physiological responses during the tilt phase. The outcomes of the test are determined based on a rigorous analysis of the observed physical alterations and their correlation with subjects’ prior symptoms during spontaneous incidents. Positive results ensue when discernible physical changes, such as diminished HR, cardiac interruptions, and reduced BP, precisely mirror patients' past symptomatic episodes. In contrast, if physical changes manifest without replicating symptoms, the results are categorized as false positives. Conversely, negative results are established when symptoms fail to align with observed physical changes or when neither symptoms nor physical alterations manifest during the test [13].

### Data preprocessing on physiological signals

Our research integrates various aspects to provide a comprehensive understanding of physiological responses during the study. To begin, the feature extraction approach entails the analysis of two distinct time periods. The initial 10-min segment positions subjects in a supine resting state, followed by a subsequent 15-min period during which they are tilted at a 70-degree angle with administered GTN. This detailed approach allows for a thorough analysis of beat-to-beat HR and BP signals.

In the context of the HUTT, the timing of VVS occurrence varies among subjects and situations. Typically, VVS manifests during the test itself, although the precise timing can differ. Some subjects experience VVS early in the HUTT window, within the first few minutes, while others encounter it later. These variations can be attributed to the unique physiological responses, medical history, and specific test circumstances of the subjects.

As part of the study, an extensive data preprocessing methodology for physiological signals was employed. This involved a thorough examination of time domain parameters of heart rate variability (HRV) and blood pressure variability (BPV). Additionally, an in-depth analysis of the frequency domain variability in heart rate and blood pressure was conducted. This multifaceted data preprocessing approach is crucial for obtaining a comprehensive understanding of the physiological responses throughout the study.

### Time domain parameters of HRV and BPV

The modulation of HR involves both the sympathetic and parasympathetic branches of the ANS. Sympathetic activity increases HR but lowers HRV, while parasympathetic activity decreases HR but increases HRV [29]. The regulation of autonomic output involves interconnected parts of the Central Nervous System (CNS). In certain situations, the vagus nerve can react abnormally, causing a sudden drop in BP, slower HR, widened blood vessels, excessive sweating, and various symptoms like dizziness, nausea, blurred vision, ultimately leading to fainting (syncope) [6–8]. Moreover, fluctuations in BP result from complex interactions between various cardiovascular systems, including central autonomic regulation, sympathetic vascular modulation, baroreflex, and humoral influences [30]. In response to PNS activation, BP is lowered through vasodilation and bradycardia to prioritize blood flow to essential organs [8]. However, in VVS, these normal responses are disrupted or magnified, leading to an excessive drop in BP and HR due to factors such as an overactive PNS, heightened blood vessel sensitivity, and abnormal autonomic signalling [10]. When it comes to properly assessing the contributions of multiple underlying regulatory mechanisms, time-domain metrics reflecting the entire variability of both HR and BP are rather indiscriminate. The study calculated the standard deviation (SD), average real variability (ARV), root mean square of real variability (RMSRV), coefficient of variation (CV), and standard deviation of real variability (SDRV) in a study of both HRV and BPV in the time domain. These indexes' formulas are listed below [31]:

1 $$\mathrm{Standard\; deviation }\left({\text{SD}}\right)=\sqrt{\frac{{\sum }_{i=1}^{n}{\left({x}_{i}- \overline{x }\right)}^{2}}{n-1},}$$

2 $$\mathrm{Coefficient\; of\; variance }\left({\text{CV}}\right)=\frac{SD}{mean} x 100\%.$$

Average real variability (ARV): Variations in absolute terms between the subsequent values:

3 $$=\frac{\sum_{i=1}^{n-1}{D}_{i}}{n-1}, where\; {D}_{i=\left|{x}_{i+1}- {x}_{i}\right|.}$$

Root mean square of real variability (RMSRV): The sequential difference between the subsequent values expressed as the root mean square:

4 $$=\sqrt{\frac{{\sum }_{i=1}^{n-1}{({D}_{i})}^{2}}{n-1}.}$$

Standard deviation of real variability (SDRV): Sequential differences between the surrounding values:

5 $$=\sqrt{\frac{{\sum }_{i=1}^{n-1}{\left({D}_{i}- \overline{D }\right)}^{2}}{n-1}.}$$

Here, x = HR, SBP, and DBP are all measured from beat to beat, $\overline{x }=$ mean for the relevant variable and n = the overall number of beats for the selected variable.

### Frequency domain variability in heart rate and blood pressure

The spectrum analysis plots the variation of spectral power of HRV and BPV as functions of frequency. The adaptable autoregressive coefficients generated with each physiological parameter were utilized to calculate the frequency spectrum in the Task Force Monitor [32]. The following parameters can be determined via spectral analysis:

LF scale 0.04–0.15 Hz [33],

HF scale 0.15–0.4 Hz [34].

6 $$\mathrm{LF\; normalized\; units}-\mathrm{LF\; n}.{\text{u}}. =\frac{LF}{LF+HF}*100,$$

7 $$\mathrm{HF\; normalized\; units}-\mathrm{HF\; n}.{\text{u}}.=\frac{HF}{LF+HF}*100.$$

Since HF + LF < 100.

The relationship and balance of both sections of the ANS can be determined via spectral analysis, however, absolute levels of HRV and BPV features are not indicators of ANS activity. The HF domain is thought to be mostly influenced by parasympathetic modulation, while the LF is primarily influenced by sympathetic modulation. The LF-to-HF ratio is used to estimate the balance of both components of the ANS’s influence on the heart.

### Missing value imputation

In our study, which encompassed data from 137 subjects undergoing HUTT, both test-positive and test-negative subjects were included. Within this group, we identified missing data in a total of 17 subjects—comprising 12 test-negative and 5 test-positive subjects. The specific missing data pertained to features referred to as systolic blood pressure variability in both supine and tilting positions [Hfnu_SBPV (high-frequency normalized power of systolic BP variability), Lfnu_SBPV (low-frequency normalized power of systolic BP variability), LFHF_SBPV (ratio of high frequency to low frequency normalized power of systolic BP variability)] and diastolic blood pressure variability in the same positions (Hfnu_DBPV (high-frequency normalized power of diastolic BP variability), Lfnu_DBPV (low-frequency normalized power of diastolic BP variability), LFHF_DBP (ratio of high frequency to low frequency normalized power of diastolic BP variability) within our dataset. Notably, the pattern of missing data we encountered was not at random. Interestingly, the same set of features exhibited missing data across all 17 subjects, suggesting a non-random and potentially systematic underlying cause for these gaps. These instances of missing data, often represented as "NAN" (Not a Number) values, can be attributed to several contributing factors, which may include cuff-related issues, variations in sensor sensitivity, technical anomalies, and signal saturation. These circumstances can lead to intermittent temporal gaps within our recorded data. It is important to highlight that these factors collectively influence the overall quality and comprehensiveness of our dataset, and they have the potential to impact subsequent analyses and interpretations. Confronted with the presence of "NAN" data during the HUTT, the medical experts chose to move forward, leveraging their clinical expertise. Following a thorough assessment of the situation, they determined that the data gap's impact on the test's reliability was minimal, taking into account considerations such as the patient's health, the test's objectives, and the potential consequences of restarting the test. This decision was bolstered by a robust 10-min supine baseline, a carefully controlled 70-degree tilt maintained for 20 min, and the introduction of a 400 µg GTN potentiation. In essence, their choice exemplified a commitment to patient-centered care, opting to avoid an early test restart in order to prioritize the well-being of the patient.

In this study, missing data were addressed using two methods: KNN imputation and mean imputation. For KNN imputation, implemented with Scikit-learn's *KNNImputer* function, missing values were determined based on the Euclidean distance to the nearest neighbors. Mean imputation involved using the mean value of each feature, calculated through Python's panda’s library, to fill in missing values. Both techniques were selected for their simplicity, efficiency, and widespread use. While KNN imputation captures underlying relationships for accuracy, mean imputation is widely adopted due to its ease of implementation [35]. By employing both methods, the study enabled performance comparison and a robust approach to managing missing data [36, 37].

### Selection of features

Three distinct methods were employed to identify the most significant features within the dataset: FI, GA, and RFE. Independently, all three techniques were utilized, retaining features chosen by the most fitting candidates. For assessing feature importance (FI), the Scikit-learn library's RF approach was adopted. Utilizing the featureimportances attribute, relevance scores were computed for each feature. The top three features were then identified for further analysis, following an averaging process.

The genetic algorithm (GA) approach utilized the LR classifier from Python's Scikit-learn module, with parameters like crossover and mutation probabilities optimized for optimal feature selection. The fitness function was tailored to prevent overfitting, selecting relevant features based on cross-validation accuracy.

In recursive feature elimination** (**RFE), the decision tree algorithm from Scikit-learn was employed. With configuration to retain three features, the iterative process recursively eliminated the least significant feature, leaving behind the remaining features for in-depth examination.

### Statistical analysis

To ascertain if test positive and test negative are subsets from the same population, the MWUT, a statistical hypothesis test, is performed. An affirmative outcome for a HUTT occurs when symptoms of syncope reoccur alongside a corresponding reduction in HR or BP. Conversely, a negative result pertains to the absence of symptom recurrence, regardless of the HR or BP changes, or when symptoms manifest without a corresponding HR or BP response. Test positivity was determined by the medical expert supervising the HUTT. If the statistical result exceeds 0.05 (P ≤ 0.05), the study rejects the null hypothesis and draws the conclusion that the two samples did not come from the same population [15].

### Machine learning classifiers

In the study, six machine learning classifiers, namely SVM, KNN, MNB, GNB, LR and RF for VVS categorization.

#### Support vector machine

The study in Support vector machine (SVM) algorithm accustomed the linear or radial basis function (RBF) kernel. C is a penalty parameter with values 0.1,1,10 and 100. Gamma is in the range of 0.1, 0.001, 0.001, and 0.0001.

#### K-nearest neighbors

The K-nearest neighbors (KNN) algorithm's power parameter for the Minkowski distance metric is denoted by "p". p and n_neighbors in this experiment had values of 1 to 10.

#### Multinomial naïve Bayes

When it comes to HUTT data, multinomial naïve Bayes (MNB) can be utilized to assess the probability of a patient experiencing VVS. Our study had fine-tuned parameter alpha values such as 0.001,0.01,0.1,0.5,1.0,10.0, and 100.0.

#### Gaussian naïve Bayes

When working with continuous data, it’s common to assume that each class's continuous values will be distributed in a Gaussian naïve Bayes (GNB). Here the range of var_smoothing was log space (0, − 9, number = 100).

#### Logistic regression

The frequency of a target attribute is forecasted using a LR. Here log space (-3,3,7) and 11 and 12 were the hyperparameters C and penalty, respectively.

#### Random forest

Due to the random selection of features, which lowers the correlation between the ensemble's trees, this strategy tends to increase the ensemble’s predictive ability. The random forest (RF) model’s hyperparameters included max_depth ranges of 2 to 10, min_samples_leaf ranges of 5 to 200, and n_estimators range of 10 to 200.

### Performance evaluation

The parameters of the models our study generated were altered using *GridSearchCV* with a fivefold cross-validation for fine-tuning all classifiers. The study employed ML algorithms, all of which were initialized with a random state value of zero. This approach aimed to ensure the reproducibility of results by generating the same sequence of random numbers in each run of the algorithms. The following formulas were used to calculate our proposed models:

8 $${\text{Accuracy}}=\frac{{\text{TP}}+{\text{TN}}}{{\text{TP}}+{\text{TN}}+{\text{FP}}+{\text{FN}}},$$

9 $$\mathrm{Recall }\left({\text{\;sensitivity}}\right)=\frac{{\text{TP}}}{{\text{TP}}+{\text{FN}}},$$

10 $${\text{Specificity}}=\frac{{\text{TN}}}{{\text{FP}}+{\text{TN}}},$$

11 $${\text{Precision}}=\frac{{\text{TP}}}{{\text{TP}}+{\text{FP}}},$$

12 $${\text{F}}1\mathrm{ \;score}=2*\frac{{\text{Precision}}*{\text{Recall}}}{{\text{Precision}}+{\text{Recall}}},$$

where true positives (TP), false positives (FP), false negatives (FN), and true negatives (TN) were all included in the calculation of the confusion matrix. Regarding subjects undergoing testing, when a subject tests positive and the model accurately categorizes them as positive, they are labeled as TP. Conversely, if a subject tests negative but is mistakenly classified as positive by the model, they are referred to as FP. In the same vein, a subject with a genuine negative test result is denoted as TN. On the contrary, if a subject who tests positive is incorrectly categorized as negative, it falls under the FN classification.

### Partial dependence plot

Partial Dependence Plot (PDP) showed the behaviors of the model and assisted in determining which characteristics had the most influence on the outcome of the decision-making process [38, 39]. To implement this XAI model, the study employed the Scikit-learn module in Python with a RF classifier, effectively fitting the model.

## Data Availability

The study’s data and resources are presently being analyzed and cannot yet be accessed by the public. We are unable to offer more details or access to the data currently due to the ongoing nature of the research.
